# The role of European health system characteristics in affecting Covid 19 lethality during the early days of the pandemic

**DOI:** 10.1038/s41598-021-03120-2

**Published:** 2021-12-09

**Authors:** Monica Giancotti, Milena Lopreite, Marianna Mauro, Michelangelo Puliga

**Affiliations:** 1 Department of Clinical and Experimental Medicine, Magna Graecia University, Viale Europa, Catanzaro, Italy; 2 Department of Economics, Statistics and Finance, University of Calabria, Calabria, Italy; 3 Department of Clinical and Experimental Medicine, Magna Graecia University, Catanzaro, Italy; 4 Institute of Management, Sant’Anna School of Advanced Studies, Pisa, Italy; 5 Linkalab Computational Laboratory, Cagliari, Italy

**Keywords:** Health care economics, Health policy

## Abstract

This article examines the main factors affecting COVID-19 lethality across 16 European Countries with a focus on the role of health system characteristics during the first phase of the diffusion of the virus. Specifically, we investigate the leading causes of lethality at 10, 20, 30, 40 days in the first hit of the pandemic. Using a random forest regression (ML), with lethality as outcome variable, we show that the percentage of people older than 65 years (with two or more chronic diseases) is the main predictor variable of lethality by COVID-19, followed by the number of hospital intensive care unit beds, investments in healthcare spending compared to GDP, number of nurses and doctors. Moreover, the variable of general practitioners has little but significant predicting quality. These findings contribute to provide evidence for the prediction of lethality caused by COVID-19 in Europe and open the discussion on health policy and management of health care and ICU beds during a severe epidemic.

## Introduction

The new coronavirus (SARS-CoV-2 or COVID-19) outbreak appeared in Wuhan, China in December 2019 and rapidly progressed in Europe becoming an urgent concern^1–3^. Despite the response activities, the epidemic has become widespread and the mortality in some European countries has been very high^4^. Currently, the pandemic has not yet ended (March 2021), and many authoritative estimations suggest a possible development with catastrophic consequences in terms of structural inequalities of income, health, and education, many from middle-income countries^5–7^. One of the most relevant factors that could dampen the devastating power of the epidemic could be the loss of lethality of the virus^4,8^, expressed as the ratio between the deaths and the infected people^9^. For these reasons, the factors influencing the COVID-19 lethality are still undergoing investigation with many studies mainly focused on local factors and health conditions.

Several papers have especially underlined the role of environmental factors in accelerating SARS-CoV-2 spread and its lethality including the chronic exposure to air pollution that might led people to be more susceptible to the COVID-19 disease, in turn leading to an increase of COVID-19 spread and its lethality^10–13^.

Focusing on the relationship between the number of reported cases and the weather variables in some regions, other studies analyzed the effects of the climate change such as temperature and humidity on the transmission of COVID-19 epidemic. The researchers demonstrated that weather variables such as temperature and humidity are essential in predicting the mortality rate of COVID-19^14^.

A large set of analyses identify the socio-demographic variables, such as *age* and *health status* the most correlated with Covid-19 lethality: people under a growing risk are in general aged over 70 years, are immunocompromised or with specific chronic medical conditions^15–19^.

Finally, health factors (e.g., national health expenditures, health infrastructure, healthcare personnel) are also particularly important^20,21^. Recent studies showed that exceeding the health capacity leads to an increase in the mortality/case ratio^22^: one of the main factors implicated in the COVID-19 deaths is the surge of cases that depleted hospital resources^23^.

Many resources are required to adequately treat a critically ill patient with COVID-19: an intensive care unit (ICU) bed with a full-featured ventilator, personal protective equipment (e.g., isolation gowns, N95 respirators, gloves, etc.), and adequate hospital staffing (doctor and nurses), in a perspective of quality care maximization during a burden disease^23^.

From this point of view, most European countries have been facing a health emergency that were not ready to address for the lack of human and structural resources. This was the consequence of restrictive health policies adopted by the main European countries in response to Organization for Economic Co-operation and Development (OECD) suggestions^24^.

Following those advices, the number of hospital beds was reduced, greatly affecting the mortality rate: There is large evidence that the countries with the highest hospital beds capacity were the ones that had the lowest numbers of deaths^23^. For example, in Germany, despite the OECD suggestions of a tightening health policy as best practice, the global number of hospital beds was growing before the coronavirus outbreak^24^. This measure contributed to improve the ability of the German health system to cope with the coronavirus spread. Indeed, according to the OECD statistics, Germany with 8 hospital beds for every thousand people is at the top rank for an optimal bed management, followed by France 5.9 (from 6.4), Italy with 3.1 hospital beds for every thousand people (in 2013 it was 3.5), Spain 3 (from 3.2) and Great Britain 2.5 (from 3 that were in 2013). In the first 40 days of the COVID-19 Germany recorded a very low death cases with respect to the diagnosed cases (37.000 cases and 206 deaths) much less than other European countries. For example, in Italy the infected were 74.000 and 7500 the victims, while in Great Britain there have been 8600 affected, and 652 deaths, 3 times those in Germany^25^. The only OECD country with performances better than Germany was Japan where there are 13 beds per thousand inhabitants. Japan, in fact, recorded few COVID-19 infections at the beginning of the epidemic, just over 1300 cases, but, in proportion, even less victims (45). In summary, this scenery could be explained by the different countries’ response in managing the coronavirus emergence: recent studies show that the hospital capacity is crucial in reducing the deaths of infected people^26,27^.

Other studies report that general practitioners (GPs) represent an important resource of a public health system^28^. The COVID-19 outbreak has refocused attention on their role. They contributed in different ways in the management of the novel coronavirus. In many European countries, GPs were at the forefront of tackling the spread of the virus. They are both gatekeepers and health promoter empowering the community to build a firewall against the deadly virus^29,30^. Even in hospital-oriented towards the health services, where the family medicine system is not yet fully implemented (as Wuhan), GPs helped in blocking the viral transmission by monitoring people at designated checkpoints in airports, railway stations and highways where they verified personal information and conducted health checks for travelers, reporting suspected cases to hospitals for urgent follow-up^28^.

Another important leading cause of Covid-19 lethality is represented by economic factors. In this sense, a variable under investigation is represented by the GDP. Recent studies seem to observe a lower rate of Covid-19-death cases in countries with higher GDP (i.e., Luxemburg)^31^. The opposite was found for countries that exhibit low levels of GDP per capita (i.e., Ukraine, Bulgaria, and Romania)^31^: people living in low-income European countries might suffer of poor health for the scarce access to health services due to their lower income^31,32^. These factors might be exaggerated during the periods of severe crises, negatively affecting the less developed regions^31,32^.

Starting from these points and given the growing number of daily reported deaths during the Covid-19 outbreak^25^, it becomes crucial to evaluate the lethality connected to SARS-COV2, investigating the leading factors that may affect it.

This study aims to contribute to this research field analyzing the role of health system characteristics in affecting Covid 19 lethality during the early days of the pandemic. In particular, we use an explorative approach guided by the following research question: which are the health systems’ key-driver variables that play a crucial role in predicting lethality? Using the Random Forest regression, and for the purpose of a robust analysis other Machine learning methods (ML), we measured the importance of the variables inserted in the model (also called “features”) in predicting the *target variable* lethality at different time horizons. As a further robustness check we showed the superior performance of random forest regression with respect to Principal Component Analysis (PCA) and other machine learning techniques (ML) such as AdaBoost and Lasso.

This analysis aims to present a scenery of the countries’ health systems performance and their preparedness when no specific measures have been yet taken to mitigate the coronavirus impact.

## Results

**Figure 1:**
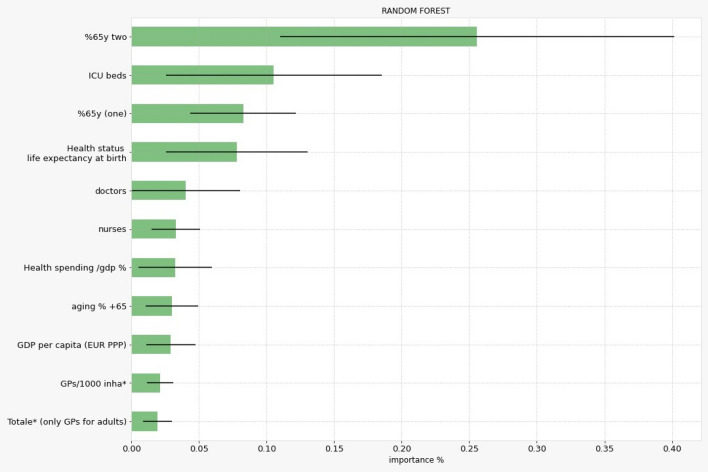
The relative importance of the variables for the best regression methods (Random Forest model): the bars show the interquartile range (IQR) centered on the median value.

Using the random forest regression, we screened the most relevant variables related to lethality during the first 40 days of the coronavirus outbreak. We consider the outcome of lethality at 10, 20, 30 and 40 days since the first fatal case of COVID-19 registered in each country as a robustness check of our results. Moreover, to obtain a summary of the distribution of the importance score of the variables inserted in the Random Forest regression we use the median as a central measure and the interquartile range (IQR) as a dispersion measure. The Fig. 1 reports in vertical lines the error bars representing the IQR (the extremes of the error bars are respectively Q1 as 25th percentile and Q3 as the 75th percentile) centered on the median value. The IQR results confirm a robust ordering of the ranking list after two careful statistical tests have been run: (a) the Kruskal-Wallis non parametric H test for the medians of a population showing that the medians of the rankings are statistically different; (b) the Wilcoxon post hoc pairwise test (Tables S1, S2, S3 that show the results of the pairwise comparison for different sample sizes are available in the file of supplementary materials) that explores the pairwise difference among the medians value and it confirms that are all statistically significant.

The results obtained with Random Forest regression, are shown in Fig. 1.

The variables were ordered with respect to their importance in the lethality prediction with values in %. At the top rank of the Fig. 1 we have the most relevant variables.

Specifically, a low value of the IQR (the importance of each variable at different time frames 10, 20, 30, 40 days) shows that the ICU beds per 1000 inhabitants—one of the most important indicators of the health infrastructure—play a crucial role (a constant effect on lethality) in explaining the lethality at the beginning of the coronavirus spread. This confirms the existing literature that correlates different mortality rates to bed capacities at the beginning of the pandemic^23,26,27^ and it motivates our study that focus on the role of health system characteristics in predicting the lethality. In fact, the ICU beds were reduced before the pandemics (as result of health reforms inspired to managerialism^33^) and the cuts created a shortage of ventilators and other lifesaving devices. For this reason, in several countries the hospital systems tried to keep as low as possible the number of patients in ICU beds eventually delaying their admittance causing a likely negative impact on the lethality.

However, whether examining the average effect of the variables during the study period the indicator more influent to the lethality is still the demographic variable including population age (percentage of 65 and above years old divided by 1000 inhabitants with at last two or more chronic diseases).

Its relatively large variability appears to be due to the growing effects on the lethality in the 40 days of time interval.

This result confirms that the increase in average population age and the higher prevalence of pre-existing diseases and strain in physical functioning is strictly connected with lethality for COVID-19.

Interestingly on the other hand there are also the variables of health spending and number of doctors, which are not similar in their importance. In this case investments in health expenditure compared to GDP could be a new key-driver variable that will provide the first best level of health structures in order to prioritize intervention and prevention strategies. Similarly, the number of nurses and doctors will be inversely proportional to mortality: where there are fewer nurses and doctors, the mortality rate will be higher. In summary, more investments in health infrastructure and in general in health spending which make healthcare more accessible to a broader part of the population and more doctors and nurses could translate into less virus fatalities.

Moreover, as our results have shown, the GDP is down in the ranking list. We can explain this result in the fact that without implications for social welfare the economic growth does not necessarily lead to an increase of people wellbeing.

Finally, the variable of GPs follows GDP and shows little but significant predicting quality: the role of GPs in healthcare system can help fill the gap in population health services, contributing to reduce lethality.

To test the performance of random forest regression we compare this model with PCA, Lasso, AdaBoost each method using the RMSE. Table 1 shows the main results of the Model. The RMSE is estimated with the leave one out procedure for the lethality at 10, 20, 30, and 40 days, an average is provided in the last column, clearly showing the better performance of the Random Forest with respect to the other two models (AdaBoost and Lasso). These criteria confirm the Random Forest that shows the lowest RMSE the best method to study the feature importance.

**Table 1:** RMSE on lethality for Random Forest and Lasso, computed at different time horizons (10, 20, 30 and 40 days) deaths per million inhabitants.

Model	Lethality (10)	Lethality (20)	Lethality (30)	Lethality (40)	avg RMSE
Random Forest	32	33	83	125	68
AdaBoost	34	35	100	138	77
Lasso	155	110	340	383	247
PCA	ND	ND	ND	ND	ND

The “*avg RMSE”* column is the average of the rows.

For the PCA method there is no RMSE as the technique does not reconstruct the data.

## Discussion

Investigating the factors correlated with the COVID-19 lethality is the key-question of our work to make assumptions whether national health care systems are well-equipped and in general are prepared in dealing with a global health crisis.

Previous studies had shown that ML techniques provide accurate results using epidemiological data^34–36^.

In particular, Random Forest models have been used very frequently in prediction analyses, showing high performance with respect to other models^37–40^. Recently, they have been employed to compute COVID-19 mortality or to predict the risk of mortality^37,41,42^. The data used for the analyses are in prevalence based on patients’ physiological conditions, symptoms, demographic information^41,42^, population characteristics^43^ or blood lab results and clinical data^41,44–47^.

Unlike the previously mentioned studies, the main contribution of this paper is to analyze the role of health system characteristics in predicting Covid 19 lethality during the early days of the pandemic through the usage of a Machine Learning model.

Indeed, most of the variables inserted in the model are those that describe the characteristics of each country’s health care system.

Specifically, in this paper, we analyzed the leading causes of lethality during the first 40 days of coronavirus outbreak in 16 European countries. Assessing the variables that mostly affect the lethality is crucial to provide efficient policy decisions concerning the economy of a country and its health care system. Although the variables that we selected in our study are standard in explaining the lethality we propose a novel and original study of the benefits of applying machine learning methods, and random forest model in particular, to the assessment of the variable importance, in explaining the lethality.

We find that demographic variables are the most relevant in predicting lethality, with a prevalence of the elders aged over 65 with two or more chronic diseases. This is not surprising, and the result is in line with the frequent reports that identify people of older age belonging to risky groups of Covid-19 fatalities^36,37,45,48^.

Another relevant variable especially during the first days of the spread of the epidemic in explaining the lethality is the number of beds in ICU: our results confirm that ICU capacity planning must be a first-order concern for health authorities to efficiently estimate the demand for urgent care during a pandemic^49^. This variable is followed by the healthcare spending (percentage of GDP), and the number of nurses and doctors/1000 inhabitants. In summary, more resources in health infrastructure and in general in healthcare spending make healthcare more accessible to a broader part of the population resulting into less virus fatalities. Moreover, where there are few doctors, the mortality rate is greater. These results confirm that health policy oriented to expansion of space, staff and hospital supplies are strongly recommended during a high burden disease that depletes hospital resources.

In this study there are several strong points. Firstly, using a method like the Random Forest, we obtain a model that explains the lethality for the majority of the European countries during the first weeks of Covid-19 outbreak. Secondly, during a pandemic in which it is crucial to make timely decisions to not increase the number of victims, our results mirror the main variables for lethality prediction to mitigate the spread of the virus and to reduce the pressure on the hospitals and the health system. Finally, the Random Forest technique used here has the advantage that, unlike some traditional regression models, it also could model nonlinear relationships that exist among the variables of our analysis.

Despite these strengths, there are several limitations that should be addressed.

The main limitation of the study is that we analyze, due to lacking data, only 16 European countries. However, unlike previous studies are limited in size examining just a single country^36–38,40^, our goal is to use the data from 16 European countries to analyze with ML techniques the global effect of different health system characteristics on COVID-19 lethality during the early days of the pandemic.

Another limitation is that we did not collect information on globalization indicators such as tourists or net migration that could influence the lethality rate: increasingly globalized world and health emergencies are connected through interrelated patterns^50^. In fact, higher degrees of global connectivity enable a faster spread of diseases and then an increase of lethality.

Moreover, a potential problem in the data collection of Covid-19 cases is relative to the number of cases that can be underestimated: health statistics could miss the real number of people suffering of one or more chronic disease. Despite all these data limitations, the usage of a panel of data with 16 countries allows to limit the influence of the national, and local effects.

Finally, it could be interesting to evaluate a specific healthcare policy, a detailed investigation, especially at micro level, as the aggregated variables considered here cannot be directly translated into policy instruments: more data could offer better results. We leave these points for future research.

## Methods

### Data availability statement

Our study was conducted during the first 40 days of the coronavirus outbreak by considering 16 European Countries (Austria, Belgium, Czechia, France, Germany, Greece, Finland, Ireland, Italy, Luxembourg, Netherlands, Norway, Poland, Portugal, Romania, Spain). The other European countries were excluded due to lack of data. For each country we considered the following variables: life expectancy at birth, per capita health spending at purchasing power parity (PPP), people aged over 65 years expressed as percentage of elders with respect to their age group (without chronic disease, with one chronic disease, with multiple chronic diseases), the fraction of elders above 65 years over the population, number of GPs/1000 inhabitants, number of GPs excluding pediatrics /1000 inhabitants, number of specialized GPs/1000 inhabitants, number of doctors/1000 inhabitants, numbers of nurses/1000 inhabitants, health spending as percentage of GDP, number of beds in ICU per 1000 inhabitants lethality at 10–20–30–40 days (computed as the ratio of deaths over confirmed cases from the first day a death has been reported, it is also called “*case-fatality ratio*”^25^). Data were drawn from the Eurostat database^51^ and from countries health profiles published by OECD database^52^. Number of GPs were extracted from the reports of the European observatory of health systems and policies published by the World health organization (WHO)^53^. All data were matched with the statistics of the lethality of COVID-19 provided by the Data Repository of the Center for Systems Science and Engineering (CSSE) at Johns Hopkins University^25^.

### Declaration of agreement with journal guidelines

All methods were performed in accordance with relevant guidelines and regulations and no experiments were performed on humans and/or human tissue samples. The data used in the analysis derived from public sources, and to the best of our knowledge, there are no privacy or moral issues associated with them.

### The random forest regression

Among the various machine learning models that can perform a Regression Analysis predicting COVID-19 lethality, we are interested in those that allow us to assess the importance of every variable used in the model. The model must be able not only to correctly predict levels of lethality using independent variables but also to estimate the impact of each variable on lethality. In this work we explored Random Forest regression (RF), that we compared in terms of performance with AdaBoost, Lasso and the Principal component Analysis (PCA) (for an introduction to Ensemble methods see^54^). All of these methods were built using the “leave one out” procedure on the available data. The “leave one out” procedure is a simple method that derives from the more general cross validation technique^55^. Given a sample S of size *n* a training phase is performed on a subsample S’ of size *n-1*, leaving out a single point that is used for the validation procedure. This procedure is repeated *m* times (like 1000) with different randomly extracted points. Once completed the procedure we obtain an average performance value (RMSE error) for each model. Once selected the best one we use its feature importance ranking list for all its internal variables. As the model is used to predict the lethality values at different time horizons (10, 20, 30, 40 days) we take the average of the importance score of each variable using the median value (as a robust measure of central tendency) of the 4 cases.

In summary the steps needed to replicate this analysis are the following:

1. Prepare k-independent variables that we believe they are mostly related to the Covid-19 lethality (for instance the “number of elders above 65 years with two or more chronic diseases”). There is an observation of these variables for each country and a value of lethality to predict (at 10, 20, 30, and 40 days).
2. Setup the machine learning models (we suggest to use a library such as scikit-learn of Python, or H2o with R, that implement several methods), that are able to predict the lethality (at a given time point after the initial outbreak) with a regression model
3. To improve the ability of the regression models in reproducing the data, as the number of observations is not large (one point per each country) we employ a method called “leave one out” that works, during the training phase of the algorithm, shortening the dataset of a single *random* point and repeating the regression estimate many times (1000 iterations) in order to get an average value for the predicted lethality.
4. The models performance in predicting the lethality (Random Forest, AdaBoost, Lasso, PCA) are compared by their square root in mean error (RMSE). The most accurate model is selected after a cycle of 1000 random comparisons.
5. Starting from the most accurate model we use the “feature importance” to identify the contribution of each variable to the regression prediction.
6. Finally, to summarize the data of lethality at different time spans (from 10 to 40 days) we use the median measure and the interquartile range (IQR) to capture the dispersion and to get a more robust estimate of the importance of each variable in the final ranking list.

The Random Forest, Lasso, AdaBoost, form a group of methods that differently from the PCA model respond to alternative questions, when it comes to measure the importance of each feature. The main difference is related to the following question: (a) is the model able to reproduce the data? (b) is the model able to explain the variability of each feature?

AdaBoost^56^ is an ensemble boosting technique that sequentially uses *weak learners* (usually simple decision trees that are barely better than random guessing the results) to obtain an accurate ensemble prediction. The main idea of boosting is using a first set of weak models to generate imperfect predictions that are used as “new data” to feed a second level of estimators that in turn, run after run, will make better predictions. This sequence of estimations, refined step by step, makes the AdaBoost a powerful technique to accurately fit the data, and make future predictions, but at the risk of overfitting. The RF model is based on random ensembles of decision trees, each one solving the recursive binary problem of splitting the data into interesting features (the decisions) according to a metric such as the Root Mean Square Error (RMSE) for the regression task or Cross Entropy for classification. The RMSE metrics is based on the idea that the best regression model is the one that minimizes the total square error from the actual data. The RF regression feature importance is then a measure that estimates how much each feature contributes to the minimization of the RMSE. In other words, the most important features are the ones that improve the most the ability of the model to reproduce the data.

The Lasso^57^ technique uses a penalty score applied to the coefficients of the linear regression with the goal of reducing the Root Mean Square Error (RMSE) without overfitting the data. The algorithm will reduce (shrink) the coefficients, setting several of them to zero. The level of penalty λ controls the amount of regularization and therefore the importance of each coefficient. Higher penalties mean that more coefficients are shrunk to zero, while low penalty values imply more nonzero coefficients. The importance associated with each feature is the absolute value of each regression coefficient. While the feature importance for Lasso is easy to interpret and clear the choice of the penalty λ is crucial to keep/discard the variables. Usually, this value is selected with a cross-validation methodology. The level of λ is increased until the model is correctly able to fit the training set with a lower error, and at the same time, it is not overfitting the validation data. Once the optimal penalty level has been reached the remaining coefficients of the linear regression represent with their value the importance of each associated feature. Lasso and Random Forest link the feature importance directly to the goodness of the regression models, however, while Lasso is a linear technique, Random Forest is not. The decision trees are able to correctly perform also in those situations where data do not follow a linear rule. In this sense non-linear models are able to capture more complex relationships and are to be preferred every time there is no explicit assumption can be made on the data linearity.

Minimizing variance is a trick used also in dimensionality reduction methods such as the PCA. In this case the goal is finding a “data transformation” of the dataset that preserves the majority of the total variance of the data. Feature importance will evaluate the features that carry out the majority of data variability, and from this regard, it will be relative to the data themselves, and not to the model that explains them. For this reason, despite the PCA being an attractive method for its simplicity, it does not respond to the fundamental question of which variable contributes the most to a regression model; it answers, instead, the question of which variable contributes with the largest variance. The dataset used in this paper is of limited size, having only 16 points: one per each country. Dealing with short samples with ML methods requires some extra care for the validation part: instead of the classical cross-validation technique, that divides the dataset in a training/test set, we used the “leave one out” methodology where the validation set consists of a single random point left out during every randomization. Averaging the error committed in estimating the validation point allows to check the error, and to tune the model for optimal parameters: a procedure known as “hyperparameter tuning”.

In summary, we used the four techniques AdaBoost, Lasso, PCA and Random Forest with leave one out validation to get the best model parameters, selecting the one (in our case the Random Forest) that is the most efficient in minimizing RMSE. The random forest regression is the model that better generalizes on unseen data with no overfitting. Thus, its parameters are used to estimate the feature importance associated with each variable.

## Supplementary Information

Supplementary Information.

## Data Availability

All data is available in the main text or by request to the authors.

## References

[1] Bontempi E (2020). The Europe second wave of COVID-19 infection and the Italy “strange” situation. Environ. Res..

[2] Li Q, Guan X, Wu P, Wang X, Zhou L, Tong Y, Ren R, Leung KS, Lau EH, Wong JY, Xing X (2020). Early transmission dynamics in Wuhan, China, of novel coronavirus–infected pneumonia. N Engl. J. Med..

[3] Paules CI, Marston HD, Fauci AS (2020). Coronavirus infections—more than just the common cold. JAMA.

[4] Carta MG, Scano A, Lindert J, Bonanno S, Rinaldi L, Fais S, Orrù G (2020). Association between the spread of COVID-19 and weather-climatic parameters. Eur. Rev. Med. Pharmacol. Sci..

[5] Vollmer, M., Mishra, S., & Juliette, H. Using mobility to estimate the transmission intensity of COVID-19 in Italy: a subnational analysis with future scenarios. Imperial College London, (2020).

[6] Lopreite M, Panzarasa P, Puliga M, Riccaboni M (2021). Early warnings of COVID-19 outbreaks across Europe from social media. Sci. Rep..

[7] Sirleaf EJ, Clark H (2021). Report of the independent panel for pandemic preparedness and response: Making COVID-19 the last pandemic. Lancet.

[8] Rajan, S., Khunti, K., Alwan, N., Steves, C., MacDermott, N., Morsella, A., Angulo, E., Winkelmann, J., Bryndová, L., Fronteira, I. & Gandré, C. In the wake of the pandemic: Preparing for Long COVID. European Observatory on Health Systems and Policies, Copenhagen (Denmark); (2021). PMID: 33877759. 33877759

[9] Motta Zanin G, Gentile E, Parisi A, Spasiano D (2020). A preliminary evaluation of the public risk perception related to the COVID-19 health emergency in Italy. Int. J. Environ. Res. Public Health.

[10] Pegoraro V, Heiman F, Levante A, Urbinati D, Peduto I (2021). An Italian individual-level data study investigating on the association between air pollution exposure and Covid-19 severity in primary-care setting. BMC Public Health.

[11] Copat C, Cristaldi A, Fiore M, Grasso A, Zuccarello P, Santo Signorelli S, Conti GO, Ferrante M (2020). The role of air pollution (PM and NO2) in COVID-19 spread and lethality: a systematic review. Environ. Res..

[12] Pluchino A, Biondo AE, Giuffrida N, Inturri G, Latora V, Le Moli R, Rapisarda A, Russo G, Zappala C (2021). A novel methodology for epidemic risk assessment of COVID-19 outbreak. Sci. Rep..

[13] Gupta A, Bherwani H, Gautam S, Anjum S, Musugu K, Kumar N, Anshul A, Kumar R (2020). Air pollution aggravating COVID-19 lethality? Exploration in Asian cities using statistical models. Environ. Dev. Sustain..

[14] Christophi CA, Sotos-Prieto M, Lan FY, Delgado-Velandia M, Efthymiou V, Gaviola GC, Hadjivasilis A, Hsu YT, Kyprianou A, Lidoriki I, Wei CF (2021). Ambient temperature and subsequent COVID-19 mortality in the OECD countries and individual United States. Sci. Rep..

[15] Kidd MR (2020). Five principles for pandemic preparedness: Lessons from the Australian COVID-19 primary care response. Br. J. Gen. Pract..

[16] Muniyappa R, Gubbi S (2020). COVID- 19 pandemic, coronaviruses, and diabetes mellitus. Am. J. Physiol. Endocrinol. Metab..

[17] Zhou F, Yu T, Du R, Fan G, Liu Y, Liu Z, Xiang J, Wang Y, Song B, Gu X, Guan L (2020). Clinical course and risk factors for mortality of adult inpatients with COVID- 19 in Wuhan, China: A retrospective cohort study. Lancet.

[18] Fang L, Karakiulakis G, Roth M (2020). Are patients with hypertension and diabetes mellitus at increased risk for COVID-19 infection?. Lancet Respir. Med..

[19] Patel JA, Nielsen FBH, Badiani AA, Assi S, Unadkat VA, Patel B, Ravindrane R, Wardle H (2020). Poverty, inequality and COVID-19: the forgotten vulnerable. Public Health.

[20] Fogel, R.W. Nutrition, physiological capital, and economic growth. *Pan American Health Organization and Inter-American Development Bank*. http://www.paho.org/English/HDP/HDD/fogel.pdf (2002).

[21] Fogel RW (2004). Health, nutrition, and economic growth. Econ. Dev. Cult. Change.

[22] Liu WY, Xiao G, Tchounwou PB (2020). Response to the Covid-19 epidemic: The Chinese experience and implications for other countries. Int. J. Environ. Res. Public Health.

[23] Sen-Crowe B, Sutherland M, McKenney M, Elkbuli A (2021). A closer look into global hospital beds capacity and resource shortages during the COVID-19 pandemic. J. Surg. Res..

[24] Organization for Economic Co-operation and Development (OECD). Better policies for better lives. https://www.oecd.org/about/47747755.pdf (2011).

[25] COVID-19 map. Johns Hopkins Coronavirus Resource Center. https://coronavirus.jhu.edu/map.html (2020).

[26] Sussman N (2020). Time for bed (s): Hospital capacity and mortality from COVID-19. Covid Econ..

[27] Ciceri F, Ruggeri A, Lembo R, Puglisi R, Landoni G, Zangrillo A (2020). Decreased in-hospital mortality in patients with COVID-19 pneumonia. Pathogens Global Health.

[28] Li, D. K. T., & Zhu, S. Contributions and challenges of general practitioners in China fighting against the novel coronavirus crisis. *Family Med. Commun. Health*, **8**(2)**,** (2020). 10.1136/fmch-2020-000361 PMC7103835 32257058

[29] Yin Y, Chu X, Han X, Cao Y, Di H, Zhang Y, Zeng X (2021). General practitioner trainees’ career perspectives after COVID-19: A qualitative study in China. BMC Fam. Pract..

[30] Lauriola P, Martín-Olmedo P, Leonardi GS, Bouland C, Verheij R, Dückers ML, Van Tongeren M, Laghi F, Van Den Hazel P, Gokdemir O, Segredo E (2021). On the importance of primary and community healthcare in relation to global health and environmental threats: lessons from the COVID-19 crisis. BMJ Glob. Health.

[31] Pardhan S, Drydakis N (2020). Associating the change in new COVID-19 cases to GDP per Capita in 38 European countries in the first wave of the pandemic. Front. Public Health.

[32] Drydakis N (2015). The effect of unemployment on self-reported health and mental health in Greece from 2008 to 2013: A longitudinal study before and during the financial Crisis. Soc. Sci. Med..

[33] Mauro M, Giancotti M (2021). Italian responses to the COVID-19 emergency: Overthrowing 30 years of health reforms?. Health Policy.

[34] Bellinger C, Jabbar MSM, Zaïane O, Osornio-Vargas A (2017). A systematic review of data mining and machine learning for air pollution epidemiology. BMC Public Health.

[35] Ardabili SF, Mosavi A, Ghamisi P, Ferdinand F, Varkonyi-Koczy AR, Reuter U, Rabczuk T, Atkinson PM (2020). Covid-19 outbreak prediction with machine learning. Algorithms.

[36] An C, Lim H, Kim DW, Chang JH, Choi YJ, Kim SW (2020). Machine learning prediction for mortality of patients diagnosed with COVID-19: A nationwide Korean cohort study. Sci. Rep..

[37] Cornelius E, Akman O, Hrozencik D (2021). COVID-19 mortality prediction using machine learning-integrated random forest algorithm under varying patient frailty. Mathematics.

[38] Gupta VK, Gupta A, Kumar D, Sardana A (2021). Prediction of COVID-19 confirmed, death, and cured cases in India using random forest model. Big Data Min. Anal..

[39] Iwendi C, Bashir AK, Peshkar A, Sujatha R, Chatterjee JM, Pasupuleti S, Mishra R, Pillai S, Jo O (2020). COVID-19 patient health prediction using boosted random forest algorithm. Front. Public Health.

[40] Majhi, R., Thangeda, R., Sugasi, R. P., & Kumar, N. Analysis and prediction of COVID‐19 trajectory: A machine learning approach. *J. Public Affairs*, e2537, (2020). 10.1002/pa.2537 PMC7744840 33349741

[41] Kivrak M, Guldogan E, Colak C (2021). Prediction of death status on the course of treatment in SARS-COV-2 patients with deep learning and machine learning methods. Comput. Methods Prog. Biomed..

[42] Pourhomayoun M, Shakibi M (2021). Predicting mortality risk in patients with COVID-19 using machine learning to help medical decision-making. Smart Health.

[43] Watson GL, Xiong D, Zhang L, Zoller JA, Shamshoian J, Sundin P, Bufford T, Rimoin AW, Suchard MA, Ramirez CM (2021). Pandemic velocity: Forecasting COVID-19 in the US with a machine learning & Bayesian time series compartmental model. PLoS Comput. Biol..

[44] Yan, Li, H. Zhang, Jorge Goncalves, Yang Xiao, Maolin Wang, Yuqi Guo, Chuan Sun et al. A machine learning-based model for survival prediction in patients with severe COVID-19 infection. *medRxiv*; (2020). 10.1101/2020.02.27.20028027.

[45] Karthikeyan, A., Garg, A., Vinod, P. K., & Priyakumar, U. D. Machine learning based clinical decision support system for early COVID-19 mortality prediction. *Front. Public Health*, **9**, (2021). 10.3389/fpubh.2021.626697 PMC8149622 34055710

[46] Kar S, Chawla R, Haranath SP, Ramasubban S, Ramakrishnan N, Vaishya R, Sibal A, Reddy S (2021). Multivariable mortality risk prediction using machine learning for COVID-19 patients at admission (AICOVID). Sci. Rep..

[47] Wang J, Yu H, Hua Q, Jing S, Liu Z, Peng X, Luo Y (2020). A descriptive study of random forest algorithm for predicting COVID-19 patients outcome. PeerJ.

[48] Levin, A. T., Hanage, W. P., Owusu-Boaitey, N., Cochran, K. B., Walsh, S. P., & Meyerowitz-Katz, G. Assessing the age specificity of infection fatality rates for COVID-19: systematic review, meta-analysis, and public policy implications. *Europ. J. Epidemiol.*, pp. 1–16, (2020). 10.1007/s10654-020-00698-1 PMC7721859 33289900

[49] Goic M, Bozanic-Leal MS, Badal M, Basso LJ (2021). COVID-19: Short-term forecast of ICU beds in times of crisis. PLoS ONE.

[50] Board GPM (2019). A world at risk: annual report on global preparedness for health emergencies.

[51] Eurostat statistics. Available at https://ec.europa.eu/eurostat/statistics-explained/index.php/Main_Page

[52] Organization for Economic Co-operation and Development (OECD). State of Health in the EU. Country Health Profile 2019. Available at https://www.oecd.org/health/country-health-profiles-eu.htm

[53] Kringos, D. S., Boerma, W. G., Hutchinson, A., Saltman, R. B., & World Health Organization. Building primary care in a changing Europe. World Health Organization. Regional Office for Europe, (2015). 29035488

[54] Khaled F, Mohamed MG, Eyad E (2014). Random forests: From early developments to recent advancements. Syst. Sci. Control Eng. Open Access J..

[55] Andrew Y. Ng. Preventing "Overfitting" of Cross-Validation Data. In Proceedings of the Fourteenth International Conference on Machine Learning (ICML '97). Morgan Kaufmann Publishers Inc., San Francisco, CA, USA, 245–253, (1997).

[56] Robert E. Schapire. A brief introduction to boosting. In Proceedings of the 16th international joint conference on Artificial intelligence - Volume 2 (IJCAI'99). Morgan Kaufmann Publishers Inc., San Francisco, CA, USA, 1401–1406, (1999).

[57] Muthukrishnan, R., Rohini, R. LASSO: A feature selection technique in predictive modeling for machine learning. *2016 IEEE International Conference on Advances in Computer Applications (ICACA)*, Coimbatore, India, pp. 18–20. 10.1109/ICACA.2016.7887916 (2016).

