# Supplementary material for: The role of European health system characteristics in affecting Covid 19 lethality during the early days of the pandemic
Source: Sci Rep. 2021 Dec 9;11:23739. doi: 10.1038/s41598-021-03120-2 (PMC8660820; doi:10.1038/s41598-021-03120-2)
Supplement: Supplementary file 1 — Supplementary Information. [file 41598_2021_3120_MOESM1_ESM.docx]

**The role of European health system characteristics in affecting Covid 19 lethality during the early days of the pandemic. Supplementary Materials**

Monica Giancotti^1^, Milena Lopreite^2*ⅰ^, Marianna Mauro^3ⅰ^, & Michelangelo Puliga^4^

1. Department of Clinical and Experimental Medicine. Magna Graecia University, Viale Europa, Catanzaro, Italy. E-mail: *mgiancotti@unicz.it*.
2. Department of Economics, Statistics and Finance, University of Calabria, Calabria, Italy. Institute of Management, Scuola Superiore Sant’Anna, Pisa, Italy. E-mail: [*milena.lopreite@unical.it*](mailto:milena.lopreite@unical.it).
3. Department of Clinical and Experimental Medicine. Magna Graecia University, Catanzaro, Italy. E-mail: [*mauro@unicz.it*](mailto:mauro@unicz.it).
4. Institute of Management, Sant’Anna School of Advanced Studies, Pisa, Italy. Linkalab Computational Laboratory, Cagliari, Italy. Email: [*michelangelo.puliga@linkalab.it*](mailto:michelangelo.puliga@linkalab.it)*.*

**Statistical tests**

We follow in this paragraph the suggestions of the book of non-parametric statistics of Weaver et al. (2017), where the authors illustrate, among others, the case of an analysis based on medians and the pairwise comparison with the post-hoc tests. The goal is studying the robustness of the ranking list of the COVID-19 lethality variables out of the Random Forest model (see the main paper for details).

We first performed the Kruskal-Wallis non parametric H test to assess the differences among the group of medians. Here the medians are computed on the simulated ranking data (importance of a variable). We found that the hypothesis of all medians coming from a single distribution can be rejected with a large confidence:

statistics=1999.6488990238468, pvalue < 0.000001

We proceeded then to compute if two paired variables have statistically different medians. We will use a pairwise post-hoc test, the Wilcoxon signed rank test, selected for its non – parametric requirements that relax the need to know in advance which statistical distribution will follow our data. The p-value correction for multi-comparison is the strict Bonferroni criteria.

The test is sensitive to the sample size, with larger samples having a reduced probability to give wrong non-significant results.

In the following Tables (Table S1, Table S2, Table S3) we report the post hoc Wilcoxon test (with the stringent Bonferroni p-value correction) when the computed statistics derive from small, medium and large paired samples (50, 500 and 2000 points per sample). Increasing the size of the sample the tests become more accurate.

Analyzing the last Table S3 that shows the results for large sample (2000 sample size) we notice that the null hypotheses in all cases (a pair of two variables have the same median value) can be rejected above a confidence level of significance equal to 0.95 (p-value <0.05). Thus, we conclude that the ranking list of variables presented in the main paper is statistically significant and robust for the statistical tests.

Table S1. Wilcoxon pairwise test with 50 points samples per variable (with ** indicating the non-significant variables)

Table S2. Wilcoxon pairwise test with 500 points samples per variable ( with ** the non-significant variables)

Table S3. Wilcoxon pairwise test with 2000 points samples per variable (with ** the non-significant variables)

The list of variables is explained here:

| %65-I | %65y (one) |
| --- | --- |
| %65-II | %65y (two) |
| GDP | GDP per capita (EUR PPP) |
| Gps | General Practioners/1000 inhab |
| HS | Health spending (gdp %) |
| LE | Health status - life expectancy at birth |
| GPad | Total* (only GPs for adults) |
| %65 | Aging +65 y(%) |
| Nurs | Nurses |
| Docs | Doctors |
| ICUb | Intensice Care Unit beds |

**References**

Weaver, K.F., Morales, V., Dunn, S.L., Godde, K. and Weaver, P.F. (2017). Parametric versus Nonparametric Tests. In An Introduction to Statistical Analysis in Research (eds K.F. Weaver, V. Morales, S.L. Dunn, K. Godde and P.F. Weaver). https://doi.org/10.1002/9781119454205.ch4
